# Assessment of Full-Eye Response to Osmotic Stress in Mouse Model In Vivo Using Optical Coherence Tomography

**DOI:** 10.1155/2015/568509

**Published:** 2015-09-30

**Authors:** Yang Ni, Baisheng Xu, Lan Wu, Chixin Du, Bo Jiang, Zhihua Ding, Peng Li

**Affiliations:** ^1^State Key Lab of Modern Optical Instrumentation, Department of Optical Engineering, Zhejiang University, Hangzhou, Zhejiang 310027, China; ^2^Department of Ophthalmology, First Affiliated Hospital, College of Medicine, Zhejiang University, Hangzhou, Zhejiang 310027, China

## Abstract

NaCl based solutions were applied as osmotic stress agents to alter the hydration state of the mouse eye. Full-eye responses to these osmotic challenges were monitored in vivo using a custom-built optical coherence tomography (OCT) with an extended imaging range of 12.38 mm. Dynamic changes in the mouse eye were quantified based on the OCT images using several parameters, including the central corneal thickness (CCT), the anterior chamber depth (ACD), the crystalline lens thickness (LT), the cornea-retina distance (CRD), the iris curvature (IC), and the lens scattering intensity (LSI). Apparent but reversible changes in the morphology of almost all the ocular components and the light transparency of the lens are exhibited. Particularly, the ocular dehydration induced by the hypertonic challenges resulted in a closing of the iridocorneal angle and an opacification of the lens. Our results indicated that the ocular hydration is an important physiological process which might be correlated with various ocular disorders, such as dry eye, cataract, and angle-closure glaucoma, and would affect the biometry and imaging of the eye. OCT uniquely enables the comprehensive study of the dynamic full-eye responses to the ocular hydration in vivo.

## 1. Introduction

The ocular hydration is an important physiological process. The process might have a significant correlation with various ocular disorders, such as dry eye [1–3], cataract [4–8], and glaucoma [9–11]. It may also affect the accuracy of the measuring tools in ophthalmology [12, 13]. Intensive efforts have been dedicated to the characterization and evaluation of the fluid transport mechanism [14–17] in ocular. The early studies were mainly based on the in vitro experiments and focused on a single component (cornea, lens, etc.) [18–20]. Ruberti and Klyce examined a trans-endothelial fluid transport system in response to NaCl osmotic challenges in the excised rabbit cornea using an automatic scanning specular microscope for corneal thickness measurement [18]. Marcantonio et al. investigated the effect of osmotic stress on lens opacification and crystallin loss using the organ culture of bovine lenses [19]. Optical coherence tomography (OCT) is a powerful noninvasive, three-dimensional (3D), real-time imaging modality with high axial resolution. As the advance of long imaging range, OCT is capable of full-eye imaging in mouse [21–24] and even in human with VSCEL swept source [25]. OCT has been used for noninvasive monitoring and quantification of diffusion of different analytes in sclera and cornea in vivo [26, 27]. Hosseini et al. reported an in vivo study of the corneal response to the dehydration stress after topical administration of hypertonic agents and prolonged surface evaporation of the cornea [28]. The osmotic stress would lead to a fluid transport between the cornea and the aqueous humor. Consequently, dynamic changes in intraocular pressure (IOP), crystalline lens, and retina should also be exhibited. Therefore, quantitatively assessing the full-eye response to osmotic stress in vivo would be of great importance to comprehensively understanding the influence of the ocular hydration.

In this pilot study, osmotic stress was induced by topical administration of the drops of NaCl based stress agents in mouse model in vivo. The dynamic changes of the cornea, the crystalline lens, the retina, and the iris were monitored simultaneously using a custom-built OCT system with an extended imaging range and high resolution. The feasibility of characterizing the full-eye response to osmotic challenge was demonstrated in vivo in mouse model by OCT.

## 2. Materials and Methods

### 2.1. System Setup

A custom-designed SD-OCT system was built for this study, which has been detailed in [29]. Briefly, a superluminescent diode (SLD) with a central wavelength of 850 nm and a spectral bandwidth of 100 nm was used as a broadband light source, offering a measured axial resolution of ~4.3 *μ*m in air. In the sample arm, an objective lens (focal length of 75 mm) was used to focus the probing light beam on the sample, yielding a measured lateral resolution of ~40 *μ*m. The spectral interference fringe between the light from the reference and sample arms was recorded by a high-speed spectrometer, equipped with a high-speed line scan CMOS camera (Basler, Ahrensburg, Germany, Sprint spL4096-140k) providing 70 kHz line scan rate. The spectrometer had a spectral resolution of 0.029 nm providing a measured imaging range of ~6.19 mm in air at half Fourier space. To fully utilize the output Fourier space for imaging, we employed a full-range complex method by utilizing the X-scanner to modulate spatial interferograms and thus removed the complex conjugate ambiguity artifact [21, 30–33], delivering a ~12.38 mm maximal imaging range and a 6 dB sensitivity rolling-off within the central ~6 mm range in air. To facilitate the implementation of full-range complex method and achieve high definition in the cross-sectional images, the scanning protocol was configured with 1500 lines per frame and a B-scan rate of 46 frames per second for 3D raster scanning. During the OCT scanning, the mouse eye was positioned at the central ~6 mm range for the best imaging quality of full eye. The system dynamic range was measured at ~50 dB at the depth-position of 0.5 mm with an incident optical power of 2.5 mW in the sample.

### 2.2. Animal Protocol

Twenty-five C57BL/6 wild type mice at 16 to 18 weeks of age were obtained from the laboratory animal center at Zhejiang University (Hangzhou, China). All animals were raised and treated in compliance with the Association for Research in Vision and Ophthalmology (ARVO) Statement for the Use of Animals in Ophthalmic and Vision Research. The study was approved by the Institutional Animal Care and Ethics Committee of Zhejiang University.

Animals were all anesthetized 10 minutes before each experiment by intraperitoneal injection of 4% chloral hydrate (10 mL/kg). In the meantime, pupils were dilated with 0.5% tropicamide/0.5% phenylephrine mixed eye-agent (Mydrin-P, Santen, Osaka, Japan). The mice were placed in a cylindrical holder and mounted on a multiaxis stage in front of the scanning probe during the experiments. A crosshair scanning pattern was designed for the fine alignment between the eye axis and the scanning beam by simultaneously examining the OCT cross-sections in the horizontal and vertical meridians.

### 2.3. Study Procedure

Saline (NaCl) solutions with different concentrations served as stress agents in this study. The osmolality of the stress agents was 1000, 500, 250, and 100 mOsmol/kg (denoted as Drop-1000, Drop-500, Drop-250, and Drop-100, resp.) which were verified by an osmometer (OSA-22; Nikkiso, Tokyo, Japan). Among them, Drop-250 induces minimal changes of the ocular tissue (referring to Figure 2(a)), and it was used as the isotonic agents in this study. To induce osmotic challenges, drops of the stress agents were applied to the mouse cornea in vivo at the rate of 1 drop per 5 mins. The 25 mice were equally divided into five groups (Control and I–IV), and one eye of each mouse was randomly selected for OCT imaging, as described in Table 1. Groups I and II were designed for hypertonic challenges with different levels (250–1000/500–250 mOsmol/kg); that is, the cornea was sequentially treated with isotonic Drop-250 for 10 mins, hypertonic Drop-1000/500 for 13 mins, and isotonic Drop-250 again for 40 mins. In the meantime, OCT imaging was performed with highest frequency of 1 time/min at the hypertonic phase to follow the acute response. Group III was conducted for hypotonic challenges (250-100-250 mOsmol/kg); that is, the cornea was sequentially treated with drops of isotonic Drop-250 for 10 mins, hypotonic Drop-100 for 24 mins, and isotonic Drop-250 again for 24 mins. Group IV was optimized for monitoring the response of the iris morphology to the osmotic challenges (250-1000-100 mOsmol/kg); that is, the cornea was sequentially treated with drops of isotonic Drop-250 for 10 mins, hypertonic Drop-1000 for 20 mins, and hypotonic Drop-100 for 20 mins. Besides, all the baseline OCT measurements were performed 5 times prior to each osmotic challenges. The time range of the NaCl agent administration for Phase 2 and Phase 3 was mainly determined by the response time that the ocular tissue required to reach a steady state. In this study, the steady state is defined as the change rate of the ocular tissue has decreased to less than 1% of the initial rate.

### 2.4. Data Processing

To quantify the dynamic changes, a custom semiautomatic algorithm was developed for biometric analysis. The cross-sectional slice including the corneal vertex location was extracted from each 3D dataset at the horizontal meridian. Optical distortion in the cross-section was corrected based on Snell's principle [34], in which the refractive indices *n* of 1.40, 1.33, 1.57, and 1.33 were used for cornea, aqueous humor, crystalline lens, and vitreous cavity, respectively [35]. According to our OCT measurements, the refractive indices *n* of Drop-1000, Drop-500, Drop-250, and Drop-100 are 1.344, 1.342, 1.337, and 1.333, respectively, and the maximal difference between them is less than 0.82%. Thus, the refractive indices of ocular tissue should not change significantly in response to these osmotic agents and were assumed to be constant and independent of the hydration level in our study. Four parameters were measured along the anteroposterior (AP) axis [21, 35], including the central corneal thickness (CCT), the anterior chamber depth (ACD), the crystalline lens thickness (LT), and the cornea-retina distance (CRD). Moreover, the lens scattering intensity (LSI) and the iris curvature (IC) were measured to evaluate the optical property of the lens and the morphologic change of the iris, respectively. The definitions of the parameters were summarized in Table 2 [21, 35]. 3D OCT imaging of a commercially available model eye (OEMI-7, Ocular Instruments Inc., Bellevue, WA) was performed 5 times to validate the accuracy and the repeatability of the developed algorithm. The anterior segment parameters of the model eye were measured and matched well with the values provided by the manufacturer, as shown in Table 3.

## 3. Results and Discussions

**Figure 1:**
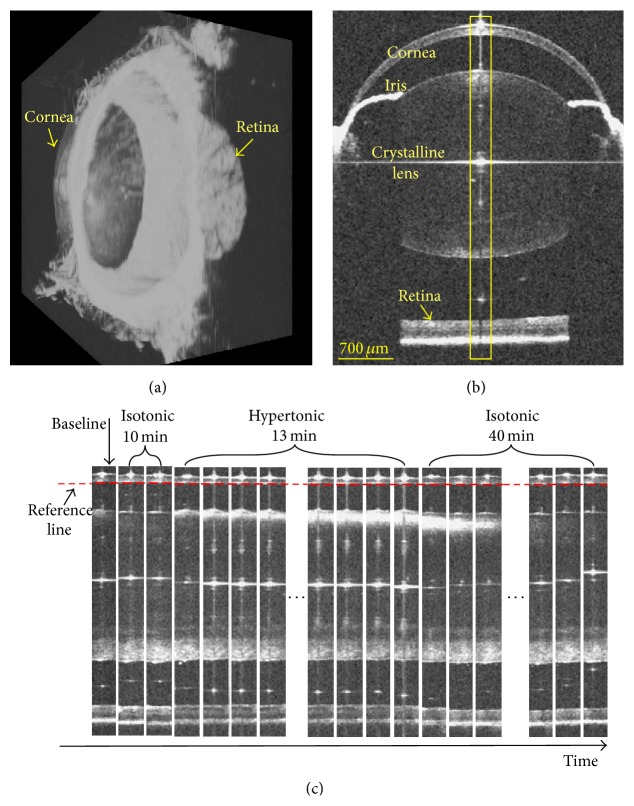
OCT full-eye imaging and dynamic response to hypertonic challenge and reversal in mouse model. (a) Three-dimensional rendering of the OCT full-eye imaging of mouse. (b) Representative OCT cross-section image in the horizontal meridian. As marked by the yellow rectangular region in (b), a serial of clips around the corneal vertex reflection line were extracted and displayed along the time dimension to demonstrate the dynamic response in (c). In the depth direction, all clips were aligned along the posterior cornea.

Figure 1(a) is a 3D rendering of the OCT full-eye imaging in the mouse model, and Figure 1(b) is a representative OCT cross-section in the horizontal meridian. As illustrated in Figure 1(b), the primary ocular components can be clearly visualized in the wide field OCT imaging, including cornea, iris, crystalline lens, and retina, based on which the biometric measurements could be readily performed using the processing algorithms. As marked by the yellow rectangular region in Figure 1(b), a serial of clips centered at the AP axis are extracted from different phases of Group I and display along the time dimension in Figure 1(c). All these clips are aligned along the posterior surface of the cornea in the depth direction. Apparently, due to the dehydration induced by the hypertonic challenge, a thinning of the cornea, decrease of ACD and CRD, and an obvious increase of the light-scattering of the anterior lens are exhibited. The reversibility of all these changes can be visualized after the return to isotonicity. In addition, it is shown that the scattering property of the anterior lens would change gradually from the surface towards the center in both the dehydration and the rehydration phases. In the transparent lens, the collagen fibers are arranged in order [36, 37]. The osmotic stress induces the water movement in the lens, and the normal distribution of fibers is changed. The disordered arrangement of fibers results in the increase of light scattering in lens and the loss of transparency [36, 37]. The gradual change of the lens scattering reveals the spatial characterization of the hydration state and ultrastructure in lens.

**Figure 2:**
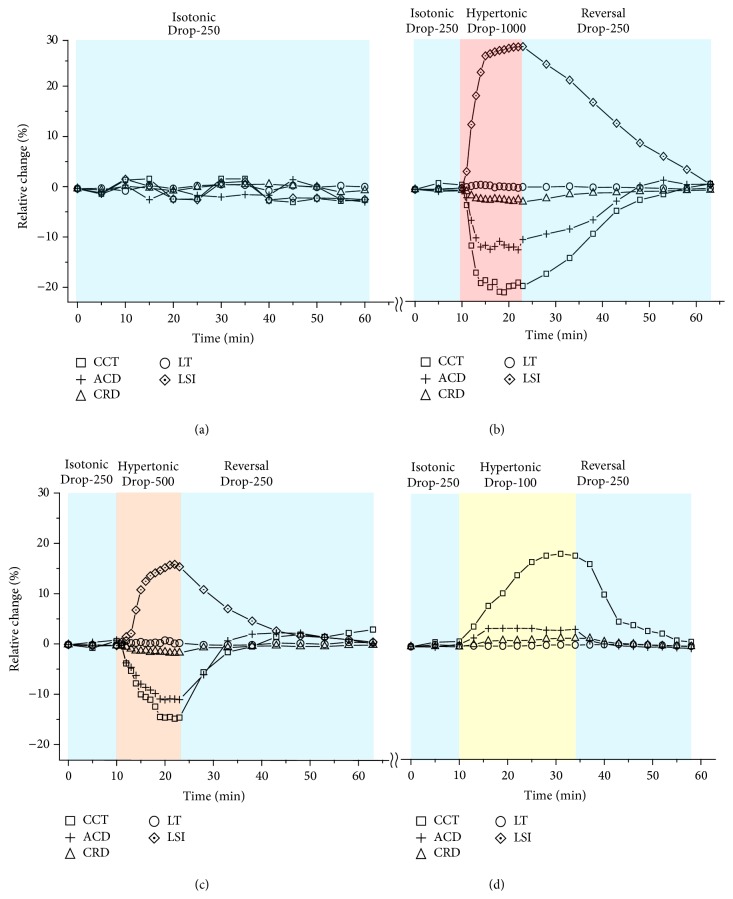
Normalized changes of cornea, lens, and retina in response to osmotic challenges. (a) Control group. (b) Hypertonic Drop-1000 challenge and reversal. (c) Hypertonic Drop-500 challenge and reversal. (d) Hypotonic Drop-100 challenge and reversal.

The dynamic responses of the cornea, the lens, and the retina to the osmotic challenges were further quantified and plotted in Figure 2. Of all the mice used in the control group and Groups I–III, the baselines (mean ± standard deviation) of the CCT, ACD, LT, and CRD are 119 ± 7 *μ*m, 404 ± 9 *μ*m, 1860 ± 14 *μ*m, and 3080 ± 18 *μ*m, respectively. To eliminate individual differences, the relative changes of all the parameters were normalized by the corresponding baseline values for each mouse. Generally speaking, the cornea, the lens, and the retina exhibited no appreciable change in the first isotonic phases, then responded with different magnitudes in the subsequent challenge phases, and gradually returned to the baselines in the reversal phases, while no meaningful change was observed in the control group (the mean standard deviation of the fluctuations <±1.5%).

The hypertonic challenge could lead to a global dehydration of the eyeball. Under the hypertonic Drop-1000 challenge (Group I), as reported in Figure 2(b), the corneal thickness (CCT) exhibited a rapid and exponential thinning of up to 20.7%. The anterior chamber depth (ACD) showed a maximal decrease of 11.5%. The relative distance between the cornea and the retina, that is, the cornea-retina distance (CRD), presented a maximal shortening of 2.3%. The crystalline lens presented no considerable change in its thickness (LT). However, the lens dehydration induced a 28.8% increase of the optical scattering (LSI), resulting in an optical opacity of the lens. The mouse eyes treated with hypertonic Drop-500 challenge (Group II) exhibited a similar changing tendency as those with Drop-1000 but with a decreased rate and magnitude, as shown in Figure 2(c). Compared with the hypertonic challenges, the hypotonic Drop-100 challenge (Group III) induced an opposite tendency at a slower changing rate of the mouse eye, as depicted in Figure 2(d). No meaningful change of the lens scattering property was observed in Group III.

**Figure 3:**
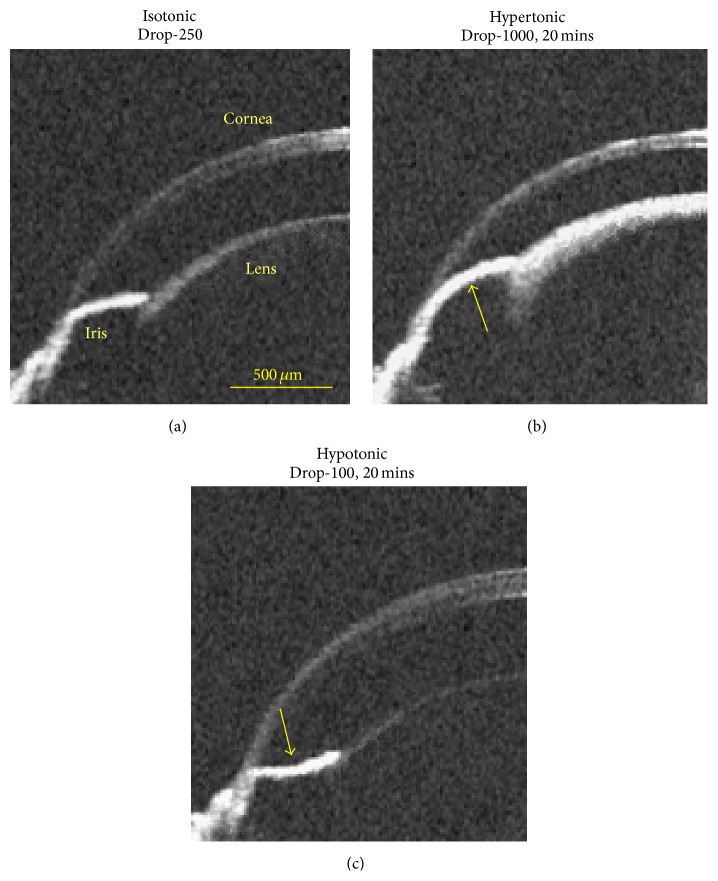
Iris response to osmotic challenges indicating the IOP change. (a) Isotonic phase. (b) Hypertonic phase at the time instant of 20 mins after the administration of Drop-1000 challenge. (c) Hypotonic phase at the time instant of 20 mins after the administration of Drop-100 challenge. The bold arrows indicate the possible direction of the IOP-induced force that acted on the iris.

Figure 3 shows the representative iris responses to the osmotic challenges. In the control group, the iris curvatures (IC) were 29.1, 30.2, and 31.8 at times 0, 30, and 60 min, respectively, and no apparent change of the iris curvature was measured. The ICs were 28.6 *μ*m, 48.2 *μ*m, and −24.6 *μ*m in the isotonic, hypertonic, and hypotonic phases, respectively. As we know, there is a close correlation between iris configuration and IOP [38]. Accordingly, most likely due to the dehydration of the cornea and anterior chamber during the hypertonic challenge, there would be a decrease of the IOP in the anterior chamber, generating a transient outward force in the posterior chamber and an outward bowing of the iris (convexity). In contrast, the hypotonic challenge would lead to a transient inward force in the anterior chamber, corresponding to an inward bowing of the iris (concavity).

In this study, it is observed from the OCT imaging that the osmotic challenges lead to reversible changes of the ocular morphology and the light scattering property of the lens. In particular, the opacification of the lens (Figure 1) and the closing of the iridocorneal angle (Figure 3) in the hypertonic challenges might be correlated with the development of cataract [4, 7, 8] and glaucoma [9–11], respectively. Thus, the hydration control is an important physiological process and might have a significant impact on almost all the ocular components, including the cornea, the iris, the lens, and the retina. OCT may offer a unique capability to quantitatively study the dynamic responses.

Rodent is one of the most frequently adopted model for improved understanding of disease pathogenesis and development of novel therapies [39, 40]. This study demonstrate that the hydration condition would significantly influence the morphological architecture of the mouse eye and may lead to a great variance in the biometric measurements. Moreover, the lens opacification appearing during the hypertonic challenge may have a great influence on the OCT image quality. As shown in Figure 1(c), the OCT intensity of the retina is apparently weak in the hypertonic phase compared with the isotonic phases. In brief, attentions should be paid to the impact of hydration control for higher measuring accuracy and imaging quality of the eye.

## 4. Conclusion

The osmotic challenge could induce a significant impact on the morphology of almost all the ocular components, including the cornea, the lens, and the retina, and the light transparency of the lens. Thus, the ocular hydration is an important physiological process, which might be correlated with various ocular disorders, such as dry eye, cataract, and glaucoma. Moreover, our results indicate that additional attentions should be paid on hydration control during the biometric measurement and fundus imaging. OCT offers a unique capability to quantitatively study the dynamic full-eye responses to osmotic stress in vivo.

## Figures and Tables

**Table 1:** Study procedures of Groups I–IV.

Group	Phase 1	Phase 2	Phase 3
Control	Isotonic (250^*∗*^)		
	60 mins, 1/5 times per min		

I	Isotonic (250^*∗*^) 10 mins, 1/5 times per min	Hypertonic (1000^*∗*^) 13 mins, 1 time per min	Isotonic (250^*∗*^) 40 mins, 1/5 times per min
II		Hypertonic (500^*∗*^) 13 mins, 1 time per min	
III		Hypotonic (100^*∗*^) 24 mins, 1/3 times per min	Isotonic (250^*∗*^) 24 mins, 1/3 times per min
IV		Hypertonic (1000^*∗*^) 20 mins, single time	Hypotonic (100^*∗*^) 20 mins, single time

^*∗*^Osmolality with unit of mOsmol/kg.

**Table 2:** Definition of parameters.

Parameters	Definition
CCT	Distance from corneal surface to corneal endothelium
ACD	Distance from corneal endothelium to anterior crystalline lens surface
LT	Distance from anterior crystalline lens surface to the posterior crystalline lens surface
CRD	Distance from corneal endothelium to the inner limiting membrane of the retina
LSI	Average backscatter intensity of the lens from the anterior capsular area to the anterior nuclear area along the AP axis
IC	Maximum perpendicular distance between the iris pigment epithelium and the line connecting the iris root and the most peripheral point of contact between the iris and lens.

**Table 3:** OCT measurement of model eye.

Parameters	OCT values ± STD (mm)	Manufacturer values ± tolerance (mm)
CCT	0.46 ± 0.01	0.55 ± 0.13
ACD	3.04 ± 0.01	3.03 ± 0.13
LT	3.85 ± 0.01	3.9 ± 0.51
